# Assessment of Women’s Awareness of the Possible Risk of Stroke Associated with the Use of Oral Contraceptives Pills in Saudi Arabia: A Cross-Sectional Study

**DOI:** 10.3390/medicina61020259

**Published:** 2025-02-03

**Authors:** Fawaz E. Edris, Rehab Ahmed A. Alasiri, Abdullah Faisal Albukhari, Mohammed Arab Sadiq, Wojood Mubarak Alahmadi, Alhanouf Radhyan Alruwaili, Nojuod Fares Alhadidi, Iman Hamid Alenezi, Hussein Talal Sabban, Abdulrahim Gari, Mamdoh Eskandar, Umme Salma, Ahmed Baker A. Alshaikh

**Affiliations:** 1 Department of Obstetrics and Gynecology, College of Medicine, Umm AlQura University, Makkah 21955, Saudi Arabia; faedris@uqu.edu.sa (F.E.E.); r1228er@gmail.com (R.A.A.A.); gari505@yahoo.ca (A.G.); 2 College of Medicine, King Abdulaziz University, Rabigh 21911, Saudi Arabia; aabdulqaderalbukhari@stu.kau.edu.sa; 3 College of Medicine, Imam Abdulrahman bin Faisal University, Dammam 34253, Saudi Arabia; mohammedsadiq8@hotmail.com; 4 College of Medicine, Vision Colleges, Riyadh 13226, Saudi Arabia; wojoodmubarak@gmail.com (W.M.A.); h78no.123@gmail.com (A.R.A.); njoodfares2@gmail.com (N.F.A.); 5 Department of Obstetrics and Gynecology, Ministry of Health, Arar 73311, Saudi Arabia; ihalenezi@moh.gov.sa; 6 GREI Consultant, King Faisal Specialist Hospital & Research Centre, Jeddah 23425, Saudi Arabia; dh.sabban@yahoo.com; 7 Obstetrics and Gynecology Department, College of Medicine, King Khalid University, Abha 62527, Saudi Arabia ; maeskandar@kku.edu.sa; 8 Department of Obstetrics and Gynecology, College of Medicine, Jouf University, Sakaka 72343, Saudi Arabia; abalshaikh@ju.edu.sa

**Keywords:** oral contraceptive pills, awareness, stroke, Saudi Arabia

## Abstract

*Background and Objectives*: One of the most widely used reversible contraceptive techniques among women of reproductive age worldwide is oral contraceptives (OCPs). Despite their widespread use, OCPs are associated with increased risks of stroke, yet the extent of awareness of these risks among women remains insufficiently explored. This study aims to evaluate the level of awareness among women resident in Saudi Arabia regarding the potential risk of stroke linked to the use of OCPs. *Materials and Methods*: A cross-sectional descriptive study was conducted targeting women of reproductive age (18–49 years). Data was collected through an online self-administrated questionnaire distributed via social media platforms, encompassing sociodemographic characteristics, OCP usage patterns, and awareness of stroke risks, perceived side effects of OCPs, symptoms of stroke, and methods to reduce stroke incidence. Statistical analysis was performed using SPSS version 23, with descriptive statistics for categorical data and chi-square tests to assess associations. *Results*: A total of 516 women participated in the study. Of these, 148, or over a quarter (28.7%), of the participants reported using oral contraceptive pills; 86, or more than half (58%), who use OCPs are not sure what type of OCPs they use; 60, or over half of the participants (40.5%), have been using for less than a year. Over two thirds of the participants (350, 67.8%) are not aware that using OCP increases the risk of stroke. The most recognized side effect of OCPs use was weight gain (38.2%), while awareness of stroke as a side effect was significantly lower (24.6%). Additionally, 62.8% of respondents identified selecting the appropriate type of contraceptive as the best way to prevent stroke while taking OCPs. *Conclusions*: this study highlights the need for increased awareness and education about the potential risk of cerebral thrombosis associated with OCP use among Saudi women. Addressing this knowledge gap through targeted educational initiatives could help mitigate the risks and improve overall public health outcomes.

## 1. Introduction

The World Health Organization (WHO) defines family planning as “the ability of individuals and couples to anticipate and attain their desired number of children and the spacing and timing of their births,” encompassing the use of contraceptive methods and therapeutic interventions for unintentional infertility [1]. Contraceptive methods include oral tablets, injectables, intrauterine devices (IUDs), sterilization, condoms, lactational amenorrhea, and natural rhythm methods [2,3]. Among these, oral contraceptives (OCs) are one of the most widely utilized reversible methods of contraception among women of reproductive age globally [4]. Oral contraceptives generally consist of estrogen and progesterone, which inhibit ovulation by suppressing the release of follicle-stimulating hormone (FSH) and luteinizing hormone (LH) from the pituitary gland. In addition to contraception, OCs offer protective benefits against several health conditions, including endometrial and ovarian cancers, dysmenorrhea, menorrhagia, iron-deficiency anemia, ectopic pregnancy, pelvic inflammatory disease, and ovarian cysts [5,6,7]. However, their use is associated with potential health risks, including an increased likelihood of venous thromboembolism, stroke, breast cancer, cervical cancer, and cardiovascular events, particularly in smokers [8,9]. Reported side effects include weight changes, nausea, abdominal bloating, breast tenderness, dermatological issues, and irregular menstrual cycles [10,11]. While the primary indication for OCs is pregnancy prevention, approximately 14% of women use them for non-contraceptive purposes, such as managing menstrual disorders and related conditions, including migraines, uterine fibroids, irregular menstruation, endometriosis-associated pain, and acne [12,13]. Stroke is a leading cause of mortality and disability worldwide, affecting approximately 15 million individuals annually and resulting in over 5 million deaths [14]. The incidence of stroke has been rising in Saudi Arabia due to significant lifestyle and environmental changes over the past two decades. Recent studies report an annual incidence rate of 29 cases per 100,000 population in the country [15,16]. Stroke, characterized as a cerebrovascular disease, is associated with high morbidity and mortality rates. Risk factors for stroke are broadly classified into modifiable factors—such as hypertension, diabetes mellitus, dyslipidemia, cardiovascular diseases, atrial fibrillation, sedentary lifestyle, obesity, smoking, and alcohol consumption—and non-modifiable factors, including age and gender [17,18,19]. These factors play critical roles in the primary and secondary prevention of stroke. An underrecognized risk factor for stroke is the use of oral contraceptives [20,21,22]. In 1969, Vessey et al. were the first to report an association between oral contraceptives and an increased risk of venous thromboembolism and cerebral thrombosis in women [23,24,25]. Subsequent observational studies have yielded mixed results, leaving the question of whether oral contraceptive use elevates stroke risk unresolved [21,26,27]. However, recent meta-analyses provide evidence suggesting a significant association between oral contraceptive use and increased risks of ischemic stroke (IS) and hemorrhagic stroke (HS). For instance, a meta-analysis demonstrated a statistically significant rise in the risk of ischemic stroke among current OC users, while another highlighted a slight increase in the risk of hemorrhagic stroke [28,29]. In Saudi Arabia, where larger family sizes are culturally valued, contraceptive use remains prevalent. The Saudi Household Survey (2018) indicated that 30.4% of women of reproductive age use contraceptives, with oral contraceptives being most commonly used by women in their twenties [30,31,32]. Oral contraceptive pills include estrogen, which suppresses follicular growth and prevents ovulation. The most frequently used oral contraceptives are combinations of ethinyl estradiol and various progestins [33,34,35]. The development of venous thromboembolism in oral contraceptive (OC) users may be linked to heightened blood coagulability, which includes changes in coagulation factors, inhibitors, fibrinolytic activity, and platelet functioning, along with structural alterations in veins and arteries, such as endothelial proliferation and altered venous flow. In contrast, the mechanisms behind arterial complications, such as myocardial infarction and stroke, are more intricate and involve increased platelet activity, fibrin deposition, higher arterial blood pressure, and modifications in glucose tolerance and lipid metabolism. Once an atherogenic lesion forms, reversing it becomes difficult due to the self-sustaining mechanisms associated with the arterial lesion [36,37,38,39]. Oral contraceptive pills that contain estrogen elevate the plasma levels of clotting factors II, VII, X, VIII, fibrinogen, and thrombin-activable fibrinolysis inhibitor (TAFI). These changes promote thrombus formation and inhibit the breakdown of clots. Among these factors, factor VII is particularly significant in clot formation. Estrogen, being a lipophilic hormone, influences gene transcription for various proteins. Although the precise mechanisms are complex and not fully understood, estrogen crosses the cell membrane of target tissues. Once inside the cytoplasm, it binds to nuclear receptors (either estrogen receptor alpha or beta). The estrogen–receptor complex then moves into the nucleus, where it binds to specific sites known as estrogen response elements. This binding enables RNA polymerase II to transcribe proteins in specific regions of DNA, activating gene transcription. However, estrogen’s influence on gene transcription is multifaceted and extends beyond the actions of the estrogen–receptor complex on DNA. Additionally, estrogen receptors affect signaling pathways, such as the MAPK and IP3 kinase pathways, which can also lead to gene expression changes. Consequently, the high levels of estrogen in oral contraceptives can contribute to clot formation, increasing the risk of ischemic stroke [40,41,42].

The present study aims to evaluate women’s awareness of the potential risk of stroke associated with oral contraceptive use. By addressing this knowledge gap, the research seeks to contribute to the growing body of evidence regarding the health implications of oral contraceptives and enhance public health awareness in Saudi Arabia.

## 2. Materials and Methods

### 2.1. Study Design

A cross-sectional descriptive study was conducted to evaluate the awareness of Saudi women regarding the potential risk of thrombosis associated with oral contraceptive pill (OCP) use.

### 2.2. Study Setting and Population

The study targeted women of reproductive age (15–49 years) residing in Saudi Arabia. Participants were recruited from five provinces: the Central, Eastern, Western, Northern, and Southern regions.

### 2.3. Inclusion and Exclusion Criteria

**Inclusion Criteria:**

- Saudi women aged 15–49 years.
- Current or past users of oral contraceptive pills.
- Women able to understand Arabic and willing to participate.

**Exclusion Criteria:**

- Women who refused to participate.
- Women with cognitive impairments or medical conditions that might confound awareness assessment.

### 2.4. Sample Size Determination

Using the WHO sample size calculator, the minimum required sample size was calculated to be 385 participants, considering a 95% confidence interval, an anticipated population proportion of 50%, and a margin of error of 5%. Accounting for a 10% non-response rate, the final target sample size was set at 424 participants. Ultimately, 516 responses were received and analyzed.

### 2.5. Data Collection Tool

Data were collected through an online self-administered questionnaire, designed in both Arabic and English to enhance accessibility and comprehension. The questionnaire was developed based on validated tools from previous studies and reviewed by a panel of medical experts to ensure content validity. A pilot study involving 25 women was conducted to test clarity and feasibility, with no major modifications required. Pilot responses were excluded from the final analysis.

The questionnaire comprised three main sections:

- **Informed Consent**: Participants provided voluntary consent before proceeding.
- **Sociodemographic Information**: Age, marital status, education level, employment status, physical activity, smoking habits, and region of residence.
- **Awareness Assessment**: Questions related to OCP usage patterns, perceived side effects, awareness of stroke risk, symptoms of stroke, and preventive strategies.

### 2.6. Data Collection Procedure

The questionnaire was distributed online via social media platforms to maximize reach. Participants were informed about the study’s objectives and their right to withdraw at any point without repercussions.

### 2.7. Data Analysis

Data were analyzed using SPSS version 23 (IBM Corporation, Armonk, NY, USA). Descriptive statistics, including frequencies and percentages, were calculated for categorical variables. Chi-square tests were performed to assess associations between sociodemographic factors and awareness levels. Statistical significance was set at *p* < 0.05. Awareness scores were categorized as follows:

- **Good Awareness**: ≥60% of the total score.
- **Poor Awareness**: <60% of the total score.

### 2.8. Ethical Considerations

The study protocol was approved by the Institutional Research Board (IRB) at Umm Al-Qura University (Approval No. HAPO-02-K-012-2024-10-2212, Date: 27 October 2024). Participation was voluntary, with confidentiality and anonymity strictly maintained. Participants were informed about their rights to withdraw at any stage without consequences.

## 3. Results

### 3.1. Participant Demographics and Characteristics

A total of 516 participants completed the study questionnaire. The vast majority (488, 94.6%) of the participants are Saudi nationals. Nearly half of participants are between 20 and 30 years old (255, 49.4%) followed by below 20 years (164, 31.8%). Concerning the education level, over half of the participants (277, 53.7%) have a bachelor’s degree. Regarding employment status, the majority (473, 91.7%) of the participants are not working. Most participants reported either occasionally (259, 50.2%) or seldom (186, 36%) engage in physical activity. The majority (489, 94.8%) of the surveyed participants are non-smokers. Over two thirds of the participants are single (352, 68.2%), while the least percentage are divorced (10, 1.9%). The survey included participants from five provinces: Central, Eastern, Western, Northern, and Southern. The highest percentage of participants live in the Central Province (224, 43.4%), followed by Western (165, 32.0%) and Eastern (31, 6.0%). Over a quarter (148, 28.7%) of the participants reported using oral contraceptive pills, as shown in Table 1.

### 3.2. The Awareness of the Risk of Stroke Associated with OCPs

**Figure 1:**
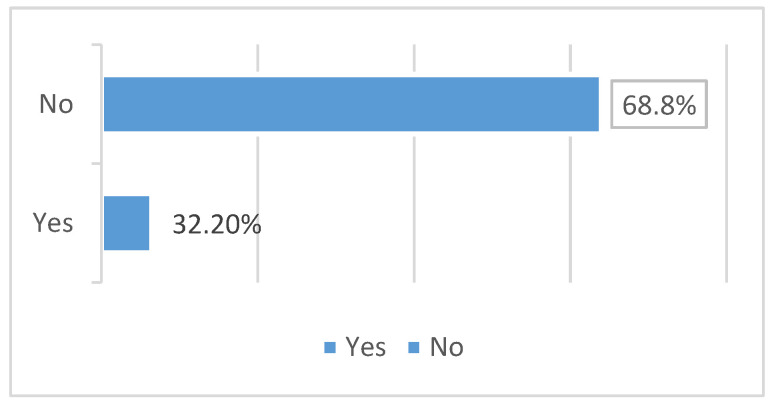
Frequency distribution of the awareness of the risk of stroke associated with OCPs use among women (*n* = 516).

Figure 1 indicates that awareness of oral contraceptive pills (OCPs) is notably high among the studied participants, with the majority selecting the combination pill. Nevertheless, a substantial proportion of individuals do not utilize oral contraceptive pills (OCPs). Weight gain is the most recognized possible adverse impact.

### 3.3. Pattern of OCPS Use

Table 2 illustrates the awareness of oral contraceptive pills (OCPs). Among the informed, the predominant kind of oral contraceptive pill utilized is the combination pill, accounting for 51 instances (9.9%). A mere 11 consumers (2.1%) are cognizant of the progestin-only tablet; 86 users (16.7%) are uncertain about the sort of oral contraceptive pill they utilize; 368 individuals (71.3%) did not utilize oral contraceptive pills (OCPs).

Among those who utilize oral contraceptive pills, the majority had been taking them for less one year. Forty-eight individuals (9.3%) have used them for a duration of 1 to 5 years. Forty-five (8.7%) and forty-three (8.3%) have utilized them for over five years. A substantial segment of 380 individuals (73.6%) questioned do not utilize oral contraceptive pills (OCPs).

### 3.4. Awareness Related to the Potential Risk of Stroke

Over two thirds of the participants (350, 67.8%) are not aware that using OCP increases the risk of stroke, as shown in Figure 1.

The most well-known possible adverse effects of OCP use among the participants were weight gain, stroke, and amenorrhea, which were reported by 197 participants (38.2%), 127 participants (24.6%), and 56 participants (10.9%), respectively. The most common symptoms of stroke reported by the participants are paralysis or weakness (356, 67%), headache with altered consciousness (38, 7.4%), and confusion (35, 6.8%).

Approximately 62.8% of participants stated that the best way to prevent stroke while taking OCPs is to select the appropriate form of contraception. About 88 (17.1%) and 38 (7.4%) of the participants, respectively, indicated routine blood pressure and weight checks, as well as maintaining a healthy weight through regular exercise and a balanced diet, as ways to prevent stroke while using OCPs as showed in Table 3.

### 3.5. Chi-Square Analysis

Table 4 presents the results of a chi-square analysis of the relationship between stroke risk scores and various personal data categories for a sample of 516 individuals. The different personal data categories that were analyzed, including nationality, age, educational level, employment status, physical activity, smoking status, marital status, province, and the number of individuals in each category who had a high or low stroke risk score. The statistical significance of the relationship between each personal data category and stroke risk score. A *p*-value (0.270) greater than 0.05 is typically considered statistically not significant. There is a statistically non-significant difference in stroke risk scores between Saudi and non-Saudi participants (*p* = 0.110, <0.05). There is a statistically non-significant trend of increasing stroke risk scores with age (*p* = 0.081, <0.05). There is a statistically non-significant difference in stroke risk scores between individuals with different levels of education (*p* = 0.767, <0.05). There is a statistically non-significant difference in stroke risk scores between individuals with different employment statuses (*p* = 0.510, <0.05). There is a statistically non-significant difference in stroke risk scores between individuals with different levels of physical activity (*p* = 0.666, <0.05). There is a statistically non-significant difference in stroke risk scores between smokers and non-smokers (*p* = 0.056, <0.05). There is a statistically non-significant difference in stroke risk scores between individuals with different marital statuses (*p* = 0.607, <0.05). There is a statistically significant difference in stroke risk scores between individuals from different provinces (*p* < 0.05). Overall, the table suggests that several personal data factors are associated with an increased risk of stroke.

## 4. Discussion

This study aimed to assess the awareness of women regarding the potential risk of stroke associated with the use of oral contraceptive pills (OCPs). According to the current study, 28% of participants either presently or formerly used OCPs, and 58% of those did not know what kind of OCP they used. The results of the study showed that women have a comparatively poor awareness of the risk of stroke, with only 32.2% of participants knowing that using OCP increases the risk of stroke. These results are less than those of a prior study that assessed women’s knowledge, attitudes, and practices around contraceptives and their side effects in the Jazan region of Saudi Arabia. It stated that around half of the individuals had either used or were now using OCPs. Overall understanding revealed that most participants knew about the OCP, with almost 95% of the women confirmed that they knew how to use them [43,44].

Furthermore, our results are marginally lower than those of comparable studies conducted in Saudi Arabia, where OCP users accounted for almost 57% of the population [45,46]. Furthermore, this is less than another survey done in Jordan, where 54% of respondents said they knew how to use the OCP [47,48]. Notably, our study was conducted through an online survey, whereas other research relied on in-person interviews. This could account for variations in OCP usage prevalence and knowledge. Furthermore, 68.2% of the study’s participants were single, suggesting that they may not have been very interested in learning about contraceptive options. Their ignorance of the issue draws attention to a serious knowledge gap in public health that must be filled.

The study revealed that weight gain is the most commonly known side effect of OCPs, reported by 38.2% of participants. In contrast, awareness of more serious potential risks, such as stroke, was significantly lower. These findings are consistent with previous research that has shown general public awareness of the more common, less severe side effects of OCPs, while underestimating the more serious health risks [49,50]. This discrepancy may be due to insufficient health education and counseling provided to women when prescribing OCPs.

The significant association between OCP use and the risk of cerebral thrombosis has been well documented in the literature. Vessey et al. first reported this association in 1969, and numerous studies have since confirmed these findings [51]. A meta-analysis by Xu et al. demonstrated a statistically significant increase in the risk of first-ever ischemic stroke among current OCP users [52]. Similarly, another meta-analysis by Xu et al. found a slight but significant increase in the risk of hemorrhagic stroke among OCP users [51]. Despite these established risks, our study indicates that awareness of these potential complications remains low among Saudi women.

Cerebral thrombosis, which involves the formation of a blood clot in the cerebral veins or sinuses, can lead to serious neurological complications, including stroke. The risk factors for cerebral thrombosis in the context of OCP use include the combination of estrogen and progestin hormones, which can increase the coagulability of blood, leading to thrombus formation [53,54]. The findings of our study underscore the urgent need for enhanced educational efforts to inform women about these risks.

The low awareness of the thrombotic risks associated with OCPs in our study population may be attributed to several factors. Cultural and social norms in Saudi Arabia may limit open discussions about contraception and its potential risks. Additionally, healthcare providers may not be sufficiently emphasizing the importance of these risks during consultations. A study conducted in Al-Qunfudah, Saudi Arabia, also found that the level of knowledge about OCPs among women of reproductive age was suboptimal, reinforcing our findings [46].

To address this gap in awareness, it is essential to implement comprehensive educational programs targeting both healthcare providers and women of reproductive age. Healthcare providers should be encouraged to discuss the full spectrum of potential risks associated with OCP use, including stroke. Public health campaigns utilizing social media platforms, which have proven effective in reaching a wide audience in Saudi Arabia, should also be employed to disseminate information about the risks of OCPs.

Our study contains a number of shortcomings. Given that women who have access to the internet and are more literate are more likely to participate, the use of an online questionnaire may have introduced selection bias. Recall bias may also result from the data’s self-reported nature. To obtain a more comprehensive knowledge of women’s awareness and perceptions of OCP hazards, future research should think about utilizing a mixed-methods approach, combining quantitative surveys with qualitative interviews. This study examined the oral contraceptive pill (OCP) users’ understanding of the risk of stroke, which had not been previously examined in Saudi Arabia.

## 5. Conclusions

This study emphasizes a significant lack of awareness among Saudi women concerning the stroke risks linked to oral contraceptive pills (OCPs). Tackling this with focused education and improved guidance from healthcare professionals is crucial to enable women to make knowledgeable choices and lower avoidable health risks. Raising awareness will improve public health results and guarantee safer contraceptive methods.

## Figures and Tables

**Table 1:** Personal characteristics of study participants, Saudi Arabia (*n* = 516).

Personal Data		No.	%
**Nationality**			
	Saudi	488	94.6
	Non-Saudi	28	5.4
**Age**			
	below 20	164	31.8
	20–30	255	49.4
	31–40	72	14.0
	41–50	19	3.7
	Above 50	6	1.2
**Educational level**			
	High school or below	183	35.5
	Diploma	44	8.5
	Bachelor’s degree	277	53.7
	Ph.D. or higher	12	2.3
**Employment status**			
	Not working	473	91.7
	Working full time	34	6.6
	Working part time	7	1.4
	Retired	2	0.4
**Physical activity**			
	Seldom	186	36.0
	Occasionally	259	50.2
	Often	71	13.8
**Smoking status**			
	Non-smoker	489	94.8
	Current smoker	21	4.1
	Ex-smoker	6	1.2
**Marital status**			
	Single	352	68.2
	Married	154	29.8
	Divorced	10	1.9
**Province**			
	Central	224	43.4
	Eastern	31	6.0
	Western	165	32.0
	Northern	43	8.3
	Southern	53	10.3
**Use of oral contraceptive pills (OCPs)**			
	Yes	136	26.4
	No	380	73.6

**Table 2:** Pattern of OCPS use among studied participants.

Items	Categories	No.	%
**Which type of OCP do you use?**	The combined pill	51	9.9
	The progesterone-only pill	11	2.1
	I don’t know the type	86	16.7
	I do not use it	368	71.3
**How long have you been using OCPs?**	Less than one year	48	9.3
	1–5 year	45	8.7
	More than 5 years	43	8.3
	Never	380	73.6

**Table 3:** Awareness of participants regarding the potential sides effect of OCPs use and the possible risk of stroke.

Awareness Items		No.	%
What potential health effects do you know are associated with the use of oral contraceptive pills?			
	Stroke	127	24.6
	Weight gain, owing to fluid retention or an anabolic effect, or both	197	38.2
	Flushing, dizziness, depression or irritability	53	10.3
	Skin changes (e.g., acne and/or an increase in pigmentation)	5	1.0
	Amenorrhoea of variable duration on cessation of taking the pill	56	10.9
	Nausea	14	2.7
	Tender breasts	14	2.7
	Headache	29	5.6
	Bloating	5	1.0
	Mood changes	5	1.0
	Headache	2	0.4
	High blood pressure	3	0.6
	Heart attack	3	0.6
	Thrombus/ clot in the lung	1	0.2
	Myocardial infarction	2	0.4
Specifically, are you aware of the risk of stroke associated with OCP use?			
	Yes	166	32.2
	No	350	67.8
What symptoms do you think are linked with a stroke?			
	Paralysis or weakness on one or both sides of the body	356	67.0
	Loss of bowel control (incontinence)	24	4.7
	Confusion, including trouble with speaking and understanding	35	6.8
	Headache with altered consciousness or vomiting	38	7.4
	Numbness of the face, arm or leg, particularly on one side of the body	19	3.7
	Trouble with seeing, in one or both eyes	5	1.0
	Trouble with walking, including dizziness and lack of co-ordination.	9	1.7
	Pain in the hands and feet that gets worse with movement and temperature changes	3	0.6
	Trouble controlling or expressing emotions.	12	2.3
	Depression	15	2.9
How do you think we can reduce the incidence of stroke when using OCP?			
	Choose the right type of contraceptive method	324	62.8
	Regular check-ups for weight, blood pressure	88	17.1
	Maintaining a healthy weight, exercising regularly, eating a balanced diet low in saturated fats and cholesterol.	38	7.4
	Use alternative forms of contraception, such as non-hormonal methods like condoms or intrauterine devices (IUDs).	38	7.4
	Manage stress	15	2.9
	Drinking enough water regularly	13	2.5

**Table 4:** Chi square analysis of stroke risk scores and personal data (*n* = 516).

Personal Data	Categories	Stroke Risk Score	*p* Value
**Nationality**			
	Saudi	159	0.270
	Non-Saudi	7	
**Age**			
	below 20	50	0.110
	20–30	78	
	31–40	29	
	41–50	9	
	Above 50	0	
**Educational level**			
	High School or below	74	0.081
	Diploma	14	
	Bachelor’s degree	78	
	PhD or higher	0	
**Employment status**			
	Not working	152	0.767
	Working full time	12	
	Working part time	2	
	Retired	0	
**Physical activity**			
	Seldom	57	0.510
	Occasionally	82	
	Often	27	
**Smoking status**			
	Non-smoker	159	0.666
	Current smoker	6	
	Ex-smoker	1	
**Marital status**			
	Single	102	0.056
	Married	59	
	Divorced	5	
**Province**			
	Central	67	0.697
	Eastern	11	
	Western	57	
	Northern	11	
	Southern	20	

## Data Availability

The datasets used or analyzed during the current study are available from the corresponding author upon reasonable request.
